# A RETROSPECTIVE COHORT STUDY ON THE RELATIONSHIP BETWEEN FRAILTY AND HEALTH CARE OUTCOMES

**DOI:** 10.1093/geroni/igae098.2752

**Published:** 2024-12-31

**Authors:** Jinmyoung Cho, Joanne Salas, Jeffery Scherrer

**Affiliations:** Saint Louis University School of Medicine, Ballwin, Missouri, United States; Saint Louis University School of Medicine, St. Louis, Missouri, United States; Saint Louis University School of Medicine, St. Louis, Missouri, United States

## Abstract

Frailty increases vulnerability for adverse outcomes in older adults. Characterizing the prevalence and distribution of frailty can help guide healthcare service decision-making and policy. This study evaluated the association between frailty and healthcare utilization and interactions by demographic characteristics. Using electronic health records (2018-2022), we conducted a retrospective cohort study with 355,266 patients ≥65 years of age who had ≥2 ambulatory office visits in separate years in the 4-year baseline period (2018-2021). The Gilbert Frailty Index (GFI) was calculated (low vs. intermediate vs. high risk) using ICD-10 codes. One-year utilization outcomes in 2022 included high outpatient clinic utilizations (OCU), inpatient (IP), emergency department (ED), and nursing home (NH) admissions. Fully adjusted log-binomial regression models were calculated overall and by race (White vs. Black), age groups, and sex. The sample was 74.5(±7.5) years of age, 57.7% female, 89.2% White, and 13.5% categorized as GFI high risk. After adjustment for covariates, GFI high risk had the highest risk for all outcomes (RR=3.31 for IP; 2.77 for ED; 4.26 for NH; 1.60 for high OCU). We observed significant interactions by race, sex, and age for each outcome. Effects of GFI high vs. low risk were larger for White (IP, ED, & high OCU), female patients (ED & high OCU), and younger patients (IP). Conversely, the effects of GFI high vs. low risk was strongest in older patients for ED, IP and high OCU. Monitoring frailty and paying attention to patient’s demographic characteristics is needed to best estimate associations between frailty and health utilization.

